# Service Use History of Individuals Enrolling in a Web-Based Suicidal Ideation Treatment Trial: Analysis of Baseline Data

**DOI:** 10.2196/11521

**Published:** 2019-04-02

**Authors:** Quincy JJ Wong, Aliza Werner-Seidler, Michelle Torok, Bregje van Spijker, Alison L Calear, Helen Christensen

**Affiliations:** 1 Black Dog Institute University of New South Wales Sydney Australia; 2 School of Social Sciences and Psychology Western Sydney University Sydney Australia; 3 Centre for Mental Health Research Research School of Population Health The Australian National University Canberra Australia

**Keywords:** internet, treatment, service use, health service, suicidal ideation, suicide, attempted, suicide, facilities and services utilization, telehealth

## Abstract

**Background:**

A significant recent innovation is the development of internet-based psychological treatments for suicidal thinking. However, we know very little about individuals experiencing suicidal ideation who seek help through Web-based services and, in particular, their previous health service use patterns.

**Objective:**

We aimed to examine service use history and its correlates among adults experiencing suicidal ideation who enrolled in a Web-based suicidal ideation treatment trial.

**Methods:**

We used baseline data of 418 individuals seeking Web-based treatment for their suicidal ideation recruited into a randomized controlled trial of a 6-week Web-based self-help program. Participants at preintervention reported demographic information, clinical characteristics, and health service use over the previous 6 months.

**Results:**

Participants had a high rate of service use in the 6 months before enrolling in the treatment trial (404/418, 96.7% of participants had contact with services). The two most common contact points were general practitioners (385/418, 92.1% of participants) and mental health professionals (295/418, 70.6% of participants). Notably, those with a previous single suicide attempt had lower odds of contact with any service than those with no attempt (odds ratio [OR] 0.21, 95% CI 0.05-0.86; *P*=.03). Those living in rural or remote areas had lower odds of contacting general practitioners (OR 0.35, 95% CI 0.13-0.91; *P*=.03) or mental health professionals (OR 0.44, 95% CI 0.23-0.83; *P*=.01) than those living in metropolitan areas.

**Conclusions:**

Individuals enrolling in an electronic health intervention trial have often received treatment from general practitioners or mental health professionals. These services can therefore play an important role in preventing the escalation of suicidal thinking. Enrollment in our Web-based treatment trial suggested, though, that face-to-face health services may not be enough. Our study also highlighted the need to improve the provision of coordinated and assertive care after a suicide attempt, as well as health service availability and utilization for those living in rural and remote areas.

**Trial Registration:**

Australian New Zealand Clinical Trials Registry ACTRN12613000410752; https://www.anzctr.org.au/Trial/Registration/TrialReview.aspx?id=364016 (Archived by WebCite at http://www.webcitation.org/6vK5FvQXy); Universal Trial Number U1111-1141-6595

## Introduction

### Background

Suicide and its precursors (suicidal thinking, plans, and attempts) are associated with individual and societal burden, making them an important public health concern [1]. Recognition that many people with suicidal thoughts do not seek help [2], coupled with rapid advances in technology and their uptake, has led to the development of internet-based cognitive behavioral treatments for suicidal thinking [3-5]. Such interventions are an important innovation in the prevention of suicidal behaviors, as they (1) target suicidal thinking (the earliest precursor to suicide), and this has the potential for important downstream effects on reducing the possibility of suicidal thoughts converting to suicidal behavior; and (2) can help to overcome several barriers to treatment, including wanting to handle the problem alone, the desire for anonymity, stigma, treatment availability, and financial and time costs [2,6]. Despite the increasing availability of e-delivery platforms targeting suicidal thoughts and behavior [7], we know very little about the historical patterns of health service use among individuals who engage with Web-based treatment programs. Understanding the previous health service use patterns of individuals who seek online help for suicidal thoughts is critically important because it can help to identify who is most likely to use such electronic health (eHealth) programs, to determine service provision gaps, and to provide new insight into the characteristics of service users to inform the design and delivery of targeted interventions.

Existing research has focused on suicidal behavior and health care use by conducting population health surveys rather than assessing users of suicide prevention services. Previous research has shown that between 31% and 57% of individuals reporting past-year suicidal ideation and 52% to 68% of individuals reporting past-year (planned) suicide attempts across high-income countries had contact with (inpatient and outpatient) mental health services in the same period [2,8]. Accessing mental health services for those with any past-year suicidal behavior (ideation, plans, and attempts) was associated with higher education, higher income, and never-married status [2]. Moreover, past-year suicidal ideation has been associated with service use after controlling for sociodemographic and clinical factors [9], whereas other research has found that being married increased and being male decreased the likelihood of service use following a suicide attempt [10]. Collectively, however, the factors associated with any form of mental health service use are not well understood. More specifically, to our knowledge, the health service use histories and correlates of adults who are seeking Web-based treatment for different suicidal behaviors have not been investigated.

### Objective

We used baseline data from a recent randomized controlled trial (RCT) conducted by our group (The Healthy Thinking Trial) that recruited adults seeking Web-based treatment for their suicidal ideation [5], to identify the patterns and correlates of participant health service use in the 6 months prior to enrolling in the study. The specific aims were to (1) examine health service use in the 6 months prior to enrollment in the study among those who experienced suicidal ideation only and those who experienced suicidal ideation and suicide attempts; and (2) describe the demographic and clinical characteristics associated with health service use.

## Methods

### Participants

We recruited participants for an RCT (registered with the Australian New Zealand Clinical Trials Registry: ACTRN12613000410752) testing the efficacy of a 6-week Web-based self-help program aimed at reducing suicidal thoughts compared with a 6-week attention-matched control program [5,11]. Between November 2013 and December 2015, we recruited community-dwelling adults via online media, which included relevant websites, popular social networking sites, and advertising on popular search engines. Interested individuals were given a link to a webpage that allowed them to provide consent and complete an online screener that verified their eligibility for the trial. The following eligibility criteria were applied: aged between 18 and 65 years; having a valid email address; having a reliable internet connection; being located in Australia; being fluent in English; having no history of a diagnosed psychotic disorder; currently experiencing suicidal thoughts; and having made no suicide attempts in the past month. These criteria were assessed by single self-report questions to which participants answered “yes” or “no” (eg, participants responded yes or no to the question “Are you currently experiencing suicidal thoughts?”). Eligible individuals were informed that the trial was not intended to replace treatment as usual. They were encouraged to continue any treatments they were already receiving or seek other treatments if they were not receiving any at the time.

### Measures

This study focused on the participant baseline data of the RCT.

#### Demographics Questionnaire

We collected the following standard demographic information: age, sex, relationship status (married or de facto; separated, divorced, or widowed; never married), education (postsecondary qualification, secondary school qualification only, no qualification), employment status (employed, unemployed, not in the labor force), and region of residence (metropolitan; regional; rural or remote). Note that rural and remote categories for region of residence were initially separate categories but were ultimately combined because of the low number of participants endorsing the remote category (n=4). We modeled the demographic categories and subcategories on previous research [8,12]. We also asked participants to indicate the number of previous suicide attempts in their lifetime.

#### Center for Epidemiologic Studies Depression Scale

The 20-item Center for Epidemiologic Studies Depression Scale (CES-D) [13] assesses the symptoms of depression over the past week. The CES-D has good psychometric properties (eg, in this study, Cronbach alpha=.87; see also Radloff [13]).

#### Generalized Anxiety Disorder Scale

The 7-item Generalized Anxiety Disorder (GAD-7) scale [14] assesses the symptoms of anxiety over the past 2 weeks. The GAD-7 scale has good psychometric properties (eg, in this study, Cronbach alpha=.85; see also Spitzer et al [14]).

#### Columbia-Suicide Severity Rating Scale

We used a self-report version of the Columbia-Suicide Severity Rating Scale (C-SSRS) [15] to indicate the severity of suicidal ideation over the past week. The C-SSRS has 5 items (answered yes/no) that assess the presence of 5 increasingly severe levels of suicidal thought (from 1 = wish to be dead to 5 = active suicidal ideation with specific plan and intent to act). A score of zero is assigned when no ideation is present. In this study, participants who reported more than one level of severity of suicidal ideation were coded with the most severe level.

#### Client Service Receipt Inventory

The original Client Service Receipt Inventory (CSRI) [16] assesses participant accommodation, employment, income, and use of health and social care services in the previous 12 months. For this study, we adapted the CSRI for the Australian context and modified it to focus on assessing whether there was any contact with the following services over the previous 6 months: general practitioner (GP), mental health professionals (including psychiatrists and psychologists), hospital services for mental health (including inpatient and outpatient services), acute services (including psychiatric crisis support team, and police or ambulance for mental health crisis), mental health helplines (eg, Lifeline), and other health services (including social worker, counsellor, and self-help group). We modeled these service use categories on previous research (eg, [2,17]).

### Procedure

We obtained ethical approval from the Human Research Ethics Committees of the University of New South Wales, Sydney (HC13117) and the Australian National University, Canberra, Australia (2012/471).

Eligible individuals were provided with an information statement, which specified the trial’s safety procedures that allowed individuals to remain anonymous in the trial if they so chose [5,11]. Subsequently, they provided consent, a valid email address, a name or nickname to register, and a phone number (nonmandatory). Participants then completed the baseline measures, which included the demographics questionnaire, CES-D, GAD-7 scale, C-SSRS, CSRI, and other measures (for details of other measures, see [5,11]), and were subsequently randomly assigned to a condition. Multimedia Appendix 1 shows the flow of participants through the trial. Multimedia Appendix 2 shows a screenshot of the Web-based self-help program aimed at reducing suicidal thoughts.

### Analyses

We used descriptive statistics to describe the full sample’s demographics and clinical characteristics and to determine the number of participants in the full sample who had contact with different health services. We also obtained these statistics for 3 subsamples (ie, those with suicidal ideation only, those with suicidal ideation and a single past suicide attempt, and those with suicidal ideation and multiple past suicide attempts). We used multivariate logistic regression models to examine variations in health service use associated with demographic variables (age, sex, relationship status, education, employment status, and region of residence) and clinical variables (depression and anxiety symptoms, suicidal ideation severity, and lifetime suicide attempt status). We conducted all analyses with IBM SPSS version 25 (IBM Corporation).

## Results

### Sample Characteristics and Health Service Use

Table 1 shows the descriptive statistics for the demographics, clinical characteristics, and past 6-month service use of the full sample and the subsamples of participants with ideation only, 1 previous suicide attempt, and multiple previous suicide attempts. A large majority of those with ideation only (186/191, 97.4%), single suicide attempts (83/89, 93%), and multiple suicide attempts (135/138, 97.8%) in our sample reported some form of contact with health services in the 6 months before enrolling in the Web-based suicidal ideation treatment trial. The two most common contact points were GPs (179/191, 93.7% of participants with ideation only had contact; 78/89, 88% of participants with a single attempt had contact; and 128/138, 92.8% of participants with multiple attempts had contact) and mental health professionals (128/191, 67.0% of participants with ideation only had contact; 60/89, 67% of participants with a single attempt had contact; and 107/138, 77.5% of participants with multiple attempts had contact). Notably, 14 of the 418 participants (3.3%) in the full sample did not have any contact with health services in the previous 6 months.

### Multivariate Logistic Regression Predicting Service Use

Table 2 shows the predictors of 6-month health service use specifically regarding contact with any service, general practitioners, and mental health professionals in the full sample. Table 3 shows the predictors of 6-month health service use specifically regarding contact with hospital services for mental health, acute services, mental health helplines, and other services in the full sample. Female participants had higher odds of contact with any service than did male participants (*P*=.03). Of note was that those who had experienced a single suicide attempt reported lower odds of contact with any service than did those with ideation only (ie, no previous suicide attempt; *P*=.03).

**Table 1:** Demographics, clinical characteristics, and 6-month service use of the sample^a^.

Characteristics		Full sample (N=418)	Ideation only (n=191)	Single attempt (n=89)	Multiple attempts (n=138)
Age (years), mean (SD)		40.64 (11.94)	41.77 (12.67)	39.93 (11.39)	39.52 (11.16)
**Sex, n (%)**					
	Male	93 (22.2)	53 (27.7)	15 (17)	25 (18.1)
	Female	323 (77.3)	138 (72.3)	74 (83)	111 (80.4)
**Relationship status, n (%)**					
	Married or de facto	160 (38.3)	82 (42.9)	31 (35)	47 (34.1)
	Separated, divorced, or widowed	72 (17.2)	30 (15.7)	16 (18)	26 (18.8)
	Never married	186 (44.5)	79 (41.4)	42 (47)	65 (47.1)
**Education, n (%)**					
	Postsecondary school qualifications	349 (83.5)	161 (84.3)	76 (85)	112 (81.2)
	Secondary school qualification only	44 (10.5)	19 (9.9)	8 (9)	17 (12.3)
	No qualification	25 (6.0)	11 (5.8)	5 (6)	9 (6.5)
**Employment status, n (%)**					
	Employed	248 (59.3)	129 (67.5)	60 (67)	59 (42.8)
	Unemployed	66 (15.8)	27 (14.1)	13 (15)	26 (18.8)
	Not in labor force	104 (24.9)	35 (18.3)	16 (18)	53 (38.4)
**Region, n (%)**					
	Metropolitan	253 (60.5)	125 (65.4)	49 (55)	79 (57.2)
	Regional	107 (25.6)	43 (22.5)	29 (33)	35 (25.4)
	Rural or remote	56 (13.4)	22 (11.5)	11 (12)	23 (16.7)
**Clinical characteristics, mean (SD)**					
	CES-D^b^	40.26 (9.62)	39.06 (8.88)	39.99 (9.69)	42.09 (10.33)
	GAD-7^c^ scale	13.27 (5.07)	12.90 (5.12)	12.78 (5.40)	14.10 (4.70)
	C-SSRS^d^ ideation severity	3.18 (1.18)	2.95 (1.17)	3.31 (1.06)	3.51 (1.11)
**Service use, n (%)**					
	Any service	404 (96.7)	186 (97.4)	83 (93)	135 (97.8)
	General practitioner	385 (92.1)	179 (93.7)	78 (88)	128 (92.8)
	Mental health professionals^e^	295 (70.6)	128 (67.0)	60 (67)	107 (77.5)
	Hospital services for mental health^f^	93 (22.2)	26 (13.6)	18 (20)	49 (35.5)
	Acute services^g^	84 (20.1)	19 (9.9)	16 (18)	49 (35.5)
	Mental health helplines	126 (30.1)	46 (24.1)	26 (29)	54 (39.1)
	Other services^h^	120 (28.7)	45 (23.6)	25 (28)	50 (36.2)

^a^In the full sample, 2 participants did not indicate their sex as male or female, and 2 participants did not provide information on region. There were no other missing data.

^b^CES-D: Center for Epidemiologic Studies Depression Scale.

^c^GAD-7: 7-item Generalized Anxiety Disorder.

^d^C-SSRS: Columbia-Suicide Severity Rating Scale.

^e^Includes psychologists and psychiatrists.

^f^Includes inpatient and outpatient services.

^g^Includes psychiatric crisis support team and police or ambulance for mental health crisis.

^h^Includes social worker, counsellor, and self-help group.

**Table 2:** Predicting 6-month service use in the full sample: contact with any service, general practitioners, and mental health professionals.

Characteristics			Any service, OR^a^ (95% CI)	General practitioner, OR (95% CI)	Mental health professionals^b^, OR (95% CI)
Age			1.02 (0.96-1.07)	1.03 (0.99-1.06)	1.00 (0.98-1.02)
**Sex**					
	Male		1 [reference]	1 [reference]	1 [reference]
	Female		4.33 (1.19-15.72)^c^	2.10 (0.89-4.94)	1.52 (0.89-2.61)
**Relationship status**					
	Married or de facto		1 [reference]	1 [reference]	1 [reference]
	Separated, divorced, or widowed		3.69 (0.42-32.66)	1.39 (0.41-4.73)	0.90 (0.48-1.69)
	Never married		2.43 (0.61-9.59)	1.00 (0.41-2.42)	1.30 (0.77-2.19)
**Education**					
	Postsecondary school qualifications		1 [reference]	1 [reference]	1 [reference]
	Secondary school qualification only		1.34 (0.14-12.55)	1.99 (0.42-9.51)	1.37 (0.62-3.06)
	No qualification		0.14 (0.02-1.14)	0.70 (0.14-3.51)	0.44 (0.17-1.10)
**Employment status**					
	Employed		1 [reference]	1 [reference]	1 [reference]
	Unemployed		1.68 (0.26-10.89)	0.74 (0.26-2.13)	1.58 (0.82-3.07)
	Not in labor force		1.48 (0.26-8.50)	0.82 (0.29-2.29)	2.08 (1.12-3.85)^d^
**Region**					
	Metropolitan		1 [reference]	1 [reference]	1 [reference]
	Regional		N/A^e^	1.32 (0.48-3.65)	1.35 (0.78-2.35)
	Rural or remote		0.35 (0.09-1.28)	0.35 (0.13-0.91)^c^	0.44 (0.23-0.83)^f^
**Clinical characteristics**					
	CES-D^g^		0.99 (0.92-1.07)	1.05 (1.00-1.10)	0.99 (0.96-1.02)
	GAD-7^h^ scale		1.11 (0.97-1.27)	1.08 (0.99-1.18)	1.05 (1.00-1.11)
	C-SSRS^i^ ideation severity		1.00 (0.61-1.66)	0.77 (0.54-1.10)	1.06 (0.86-1.31)
	**Lifetime suicide attempt**				
		Ideation only	1 [reference]	1 [reference]	1 [reference]
		Single attempt	0.21 (0.05-0.86)^c^	0.47 (0.18-1.19)	0.95 (0.54-1.69)
		Multiple attempts	0.66 (0.13-3.40)	0.88 (0.33-2.32)	1.37 (0.79-2.38)

^a^OR: odds ratio.

^b^Includes psychologists and psychiatrists.

^c^*P*=.03.

^d^*P*=.02.

^e^N/A: not applicable. All participants in the regional category (n=107) had used some form of health service. As there were no noncases, an OR was not computed.

^f^*P*=.01.

^g^CES-D: Center for Epidemiologic Studies Depression Scale.

^h^GAD-7: 7-item Generalized Anxiety Disorder.

^i^C-SSRS: Columbia-Suicide Severity Rating Scale.

**Table 3:** Predicting 6-month service use in the full sample: contact with hospital services for mental health, acute services, mental health helplines, and other services.

Characteristics			Hospital services for mental health^a^, OR^b^ (95% CI)	Acute services^c^, OR (95% CI)	Mental health helplines, OR (95% CI)	Other services^d^, OR (95% CI)
Age			0.98 (0.96-1.01)	1.00 (0.97-1.02)	0.97 (0.95-0.99)^e^	1.00 (0.97-1.02)
**Sex**						
	Male		1 [reference]	1 [reference]	1 [reference]	1 [reference]
	Female		2.14 (1.02-4.47)^f^	1.34 (0.67-2.68)	1.39 (0.76-2.55)	1.71 (0.94-3.09)
**Relationship status**						
	Married or de facto		1 [reference]	1 [reference]	1 [reference]	1 [reference]
	Separated, divorced, or widowed		0.46 (0.20-1.07)	0.68 (0.31-1.49)	0.65 (0.31-1.37)	1.24 (0.66-2.34)
	Never married		0.85 (0.48-1.50)	0.81 (0.44-1.46)	1.43 (0.86-2.40)	0.85 (0.51-1.43)
**Education**						
	Postsecondary school qualifications		1 [reference]	1 [reference]	1 [reference]	1 [reference]
	Secondary school qualification only		1.67 (0.75-3.74)	2.19 (0.99-4.82)	0.54 (0.24-1.19)	1.54 (0.76-3.13)
	No qualification		2.41 (0.90-6.49)	1.08 (0.36-3.22)	0.97 (0.37-2.56)	1.79 (0.74-4.34)
**Employment status**						
	Employed		1 [reference]	1 [reference]	1 [reference]	1 [reference]
	Unemployed		1.02 (0.49-2.11)	0.74 (0.34-1.61)	1.12 (0.60-2.09)	1.22 (0.66-2.26)
	Not in labor force		1.85 (0.99-3.43)	1.34 (0.71-2.51)	0.97 (0.53-1.75)	0.91 (0.51-1.62)
**Region**						
	Metropolitan		1 [reference]	1 [reference]	1 [reference]	1 [reference]
	Regional		1.29 (0.72-2.31)	1.20 (0.66-2.18)	1.29 (0.76-2.21)	1.08 (0.64-1.81)
	Rural or remote		0.78 (0.34-1.76)	1.07 (0.49-2.35)	1.97 (0.99-3.91)	0.97 (0.50-1.89)
**Clinical characteristics**						
	CES-D^g^		1.03 (0.99-1.07)	1.01 (0.97-1.04)	1.04 (1.00-1.07)^f^	1.02 (0.99-1.05)
	GAD-7^h^ scale		0.97 (0.91-1.03)	1.02 (0.96-1.09)	1.02 (0.97-1.08)	0.98 (0.93-1.03)
	C-SSRS^i^ ideation severity		1.34 (1.03-1.73)^j^	1.26 (0.98-1.63)	1.23 (0.98-1.53)	0.95 (0.77-1.17)
	**Lifetime suicide attempt**					
		Ideation only	1 [reference]	1 [reference]	1 [reference]	1 [reference]
		Single attempt	1.29 (0.64-2.59)	1.80 (0.86-3.79)	1.03 (0.56-1.90)	1.18 (0.66-2.13)
		Multiple attempts	2.59 (1.43-4.72)^k^	4.18 (2.22-7.85)^l^	1.54 (0.90-2.65)	1.71 (1.01-2.90)^m^

^a^Includes inpatient and outpatient services.

^b^OR: odds ratio.

^c^Includes psychiatric crisis support team and police or ambulance for mental health crisis.

^d^Includes social worker, counsellor, and self-help group.

^e^*P*=.004.

^f^*P*=.04.

^g^CES-D: Center for Epidemiologic Studies Depression Scale.

^h^GAD-7: 7-item Generalized Anxiety Disorder.

^i^C-SSRS: Columbia-Suicide Severity Rating Scale.

^j^*P*=.03.

^k^*P*=.002.

^l^*P*<.001.

^m^*P*=.046.

There were also different patterns of predictors across service categories (see Tables 2 and 3). Compared with living in the city, living in rural or remote areas was associated with lower odds of contact with a GP (*P*=.03) and lower odds of contact with mental health professionals (*P*=.01). Compared with those who were employed, those not in the labor force had higher odds of contact with mental health professionals (*P*=.02). Female participants (*P*=.04), those with more severe suicidal ideation (*P*=.03), and those who had experienced multiple suicide attempts (*P*=.002) had higher odds of contact with hospital services for their mental ill health. Not surprisingly, those who had experienced multiple suicide attempts also had higher odds of contact with acute services (*P*<.001) and other services (social worker, counsellor, and self-help group; *P*=.046). Older individuals had lower odds of contact with mental health helplines (*P*=.004), whereas those with more severe depressive symptoms had higher odds of contact with mental health helplines (*P*=.04).

## Discussion

### Principal Findings

This study aimed to examine health service use and its correlates among adults participating in a Web-based suicidal ideation treatment trial. The overwhelming majority of the sample had some form of contact with health services in the 6 months before enrolling in the Web-based suicidal ideation treatment trial, with the two most common contact points being GPs and mental health professionals. It is not possible to tell from our data whether suicidal thoughts or behaviors were addressed during contact with any of these health services because we did not assess this. That is, we cannot assume that suicidal thinking or suicide attempts were discussed during participant contact with the various health services, although they could have been. Nonetheless, the rates of service use of our sample, which was recruited for a treatment trial, were generally higher than those found in previous international [2] and Australian [8] general population studies. The relatively high rates of service use reported in our study may be due to several factors, one being the high proportion of women who participated, given that women are more likely than men to use health services (eg, [18]). Other factors that may have resulted in a higher than usual representation of service users may be the high rates of metropolitan residency, employment, and tertiary qualifications noted among the participants, which are all likely to increase geographical and financial access to services [19-22]. However, for a comparison in the Australian context, Johnston and colleagues’ [8] sample actually had similar rates of metropolitan residency and employment to that of our sample, but lower rates of women and postsecondary qualifications (see also [23]). The higher rates of women and postsecondary qualifications may thus have accounted for the greater rate of service use in our sample. Moreover, the relatively high rates of service use in our sample may also indicate a greater propensity to seek help, given that higher education has been linked to higher levels of knowledge of mental health and treatment availability [24].

The high rate of service use of our participants, together with their enrollment in the Web-based intervention, has two main implications. First, it suggests that some individuals experiencing suicidal ideation may want more help beyond in-person treatments. This may be because contact with various face-to-face services is not sufficient to meet their needs (eg, shame or discomfort disclosing suicidal thoughts or behaviors face-to-face), or they may be willing to try any intervention that may provide additional help. Future research should investigate these possibilities. Second, it suggests that the treatment trial, from which we derived this study’s sample, did not attract individuals experiencing suicidal ideation who do not normally seek help. This is despite one of the aims of the treatment trial to reach such individuals, for example, by permitting anonymous participation (see [5,11]). Another important direction for future research will be to further improve Web-based treatments to reach this group of individuals.

With respect to the correlates of health service use, the majority of results were as expected, but two findings stood out. First, we found that a previous single suicide attempt (compared with ideation only [no previous suicide attempt] status) was associated with lower odds of contact with any service. The data we collected did not allow us to determine for those participants with a previous single suicide attempt when their attempt actually occurred in their lifetime, and thus the temporal relationship with service use assessed in our study (eg, the attempt may have occurred many years prior to participating in the study, and this could explain the relatively lower odds of contact with services). However, given that a suicide attempt predicts future suicide attempts and death by suicide [25], and given that these participants with a previous single suicide attempt enrolled in our Web-based treatment trial (indicating the presence of suicidal ideation), it is of concern that these participants had a relatively lower rate of contact with any service. One reason that might account for this finding is that, in relation to a first suicide attempt, a lack of coordinated and assertive care after the attempt may lead to a general disengagement from health services [26]. Alternatively, distressing treatment experiences after the attempt may discourage health service use in general [26,27]. The second notable result from our analysis, also of concern, was that among adults participating in our Web-based treatment trial, those living in rural or remote areas had lower odds of previous 6-month contact with a GP and mental health professionals than did those living in metropolitan areas. This result is consistent with previous research demonstrating similar findings (eg, [9,21]) and, in Australia, may reflect relatively lower rates of help-seeking by individuals living in rural or remote locations, as well as the shortage of GPs and mental health professionals limiting service access in these areas [28,29]. This result continues to highlight a critical need for strategies to improve health service provision and use in rural and remote areas. In this regard, eHealth interventions may hold significant promise for delivering more accessible, evidence-based interventions to at-risk persons.

Several critical considerations emerge from this study. Given that the two most common services used by individuals with suicidal ideation before enrolling in a Web-based treatment trial were GPs and mental health professionals, it is important that such services are aware that they may be crucial intervention points to prevent the escalation of suicidal thinking. In the case of GPs, as noted above, it was not possible to ascertain why individuals in our sample consulted their GP, but it may not have been for a mental health problem or for suicidal thoughts or behaviors. Indeed, previous research has shown that only 15% of individuals who died by suicide revealed suicidal thoughts or intentions in consultations with their GPs prior to suicide [30]. This suggests that there is scope for GPs to play an important role in preventing the escalation of suicidal thinking through the routine screening of patients for suicidal thoughts and behaviors, and upskilling in terms of their capacity to treat or refer suicidal patients to appropriate services [31-33]. Notably, in a mental health service model integrating e-mental health interventions with face-to-face services, GPs would be integral in referring suitable patients to evidence-based Web-based therapies that target suicidal thinking and reduce suicide risk [5]. Our study also highlighted the need for the provision of coordinated and assertive care after a suicide attempt, especially after the first suicide attempt, to encourage engagement with health services and prevent the maintenance or escalation of suicidal thinking. Finally, as noted above, there is a need for continued emphasis on improving health service availability and use for those living in rural and remote areas who experience suicidal ideation.

### Limitations

Several limitations of our study should be considered. First, participants in our study were recruited to a trial and not users of a service. Future research should replicate our study in a sample of suicide prevention service users. Second, our sample enrolled in a Web-based suicidal ideation program after meeting inclusion criteria (and not meeting exclusion criteria) from an initial pool of 12,474 individuals who visited the registration website (see [5]). Although the exclusion of individuals was a necessity of the trial, results may have been different had these individuals been included. Third, the group of participants who did not use any services was very small, and future research should improve the recruitment of such individuals to further investigate the reasons behind their lack of service use and their decision to enroll in a treatment trial examining a Web-based intervention for suicidal ideation. Fourth, we assessed the number of past suicide attempts with a single self-report item, and future studies should improve the accuracy of identifying suicide attempts with a more in-depth assessment [34]. Fifth, our study examined cross-sectional baseline data, and we do not know the temporal relationships between the variables in our study. Sixth, as previously noted, although we assessed health service use history, we did not specifically assess whether suicidal thoughts or behaviors were addressed during contact with services. Future research should collect this information.

### Conclusions

Our study highlighted, for the first time, health service use history and its correlates among adults participating in a Web-based suicidal ideation treatment trial. Our study highlighted that these individuals had a high rate of contact with health services, particularly GPs and mental health professionals. These services can therefore play an important role in preventing the escalation of suicidal thinking. Enrollment in the Web-based treatment trial, despite this high contact with services, suggests, though, that face-to-face health services may not be enough for individuals with suicidal ideation and that they may want more help. Finally, our study highlighted the need to improve the provision of coordinated and assertive care after a suicide attempt, as well as health service availability and use for those living in rural and remote areas.

## Appendix

Multimedia Appendix 1 Participant flow diagram for the randomized controlled trial that recruited adults seeking Web-based treatment for their suicidal ideation (reproduced from van Spijker et al [5]).

Multimedia Appendix 2 Example screenshot of the Web-based self-help program aimed at reducing suicidal thoughts.

## Abbreviations

CES-D: Center for Epidemiologic Studies Depression Scale
CSRI: Client Service Receipt Inventory
C-SSRS: Columbia-Suicide Severity Rating Scale
eHealth: electronic health
GAD-7: 7-item Generalized Anxiety Disorder
GP: general practitioner
RCT: randomized controlled trial
