# Supplementary figures and images for: Service Use History of Individuals Enrolling in a Web-Based Suicidal Ideation Treatment Trial: Analysis of Baseline Data
Source: JMIR Ment Health. 2019 Apr 2;6(4):e11521. doi: 10.2196/11521 (PMC6465979; doi:10.2196/11521)

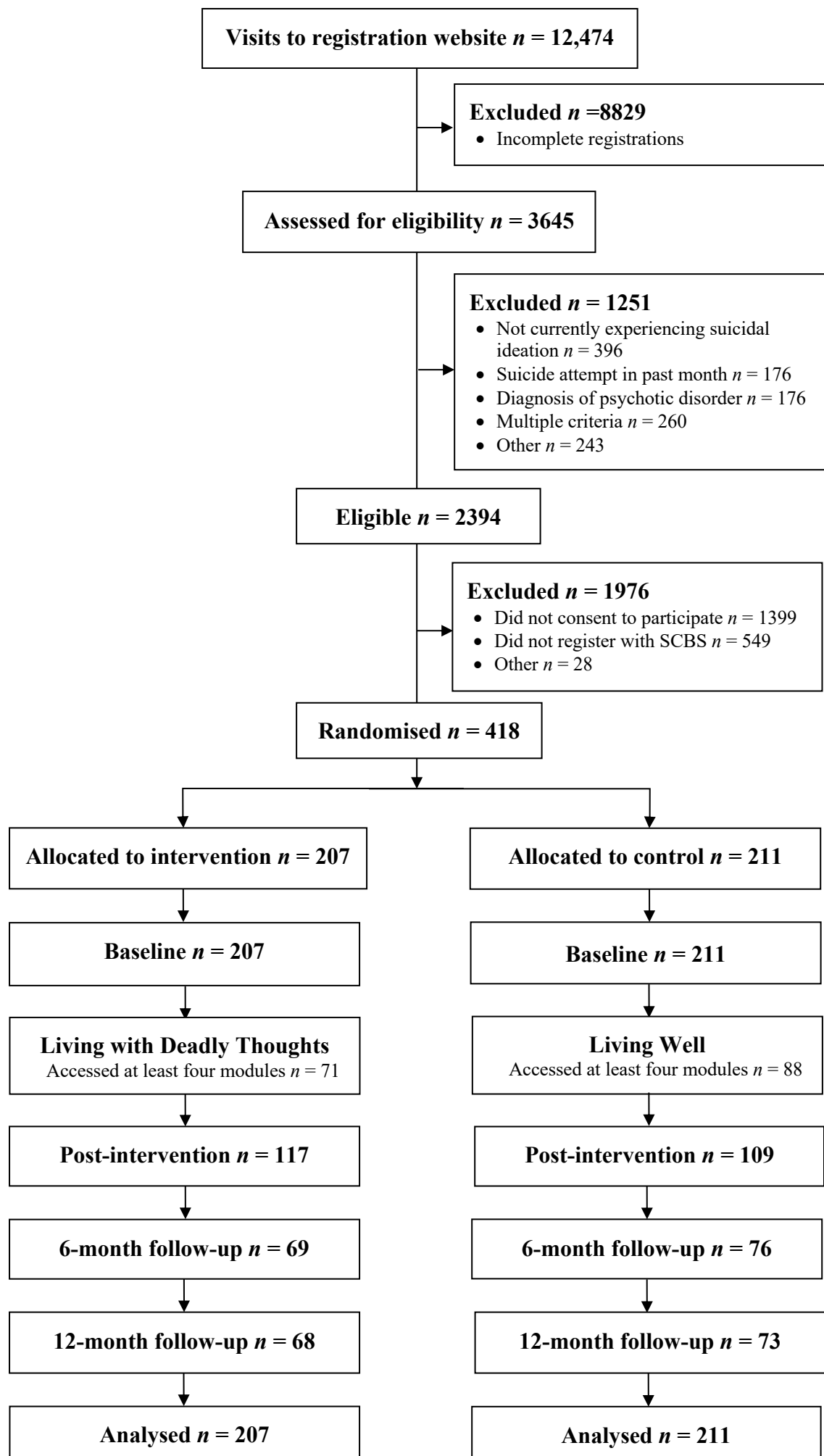

Supplement: Multimedia Appendix 1 [file mental_v6i4e11521_app1.pdf]
