# Supplementary material for: Service Use History of Individuals Enrolling in a Web-Based Suicidal Ideation Treatment Trial: Analysis of Baseline Data
Source: JMIR Ment Health. 2019 Apr 2;6(4):e11521. doi: 10.2196/11521 (PMC6465979; doi:10.2196/11521)
Supplement: Multimedia Appendix 2 [file mental_v6i4e11521_app2.pdf]

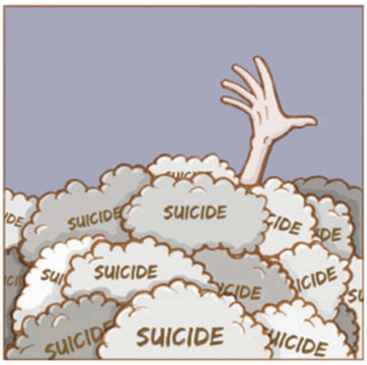

But then overtime these thoughts can become obsessive, intrusive, automatic [illegible]

Thoughts about suicide can occur frequently, ranging from a few times a day to many times throughout the day. Common thoughts are:

"What is the meaning of this life?"
"I don't have a future anymore"
"I can't live all by myself"
"Everything has failed"
"People are better off without me"

When these sorts of thoughts are repeated many times a day, they become tormenting instead of self protective. The thoughts that seemed to be helpful are now too frequent. They can intrude into your mind when you are not wanting to think about them and cause you to worry even more. Your thoughts can become so tormenting you desperately want to stop them. However, trying to control these thoughts is difficult. You may find that the more you try to stop your thoughts the more they occur. This is just how the brain works, and it happens not just for suicidal thoughts, [illegible]

copyright 2012 The Black Dog Institute
